# Dalhousie dyspnea scales: construct and content validity of pictorial scales for measuring dyspnea

**DOI:** 10.1186/1471-2431-5-33

**Published:** 2005-08-30

**Authors:** Patrick J McGrath, Paul T Pianosi, Anita M Unruh, Chloe P Buckley

**Affiliations:** 1 Departments of Psychology, Pediatrics, and Psychiatry, Dalhousie University, NS, B3H 4J1, Canada; 2 IWK Health Centre, Halifax, NS, B3K 6R8, Canada; 3 Department of Pediatrics Dalhousie University and IWK Health Centre, Halifax, NS, B3K 6R8, Canada; 4 School of Health and Human Performance and School of Occupational Therapy, Dalhousie University, 1459 Oxford Street, Halifax, NS, B3H 4R2, Canada

## Abstract

**Background:**

Because there are no child-friendly, validated, self-report measures of dyspnea or breathlessness, we developed, and provided initial validation, of three, 7-item, pictorial scales depicting three sub-constructs of dyspnea: throat closing, chest tightness, and effort.

**Methods:**

We developed the three scales (Throat closing, Chest tightness, and Effort) using focus groups with 25 children. Subsequently, seventy-nine children (29 children with asthma, 30 children with cystic fibrosis. and 20 children who were healthy) aged 6 to 18 years rated each picture in each series, using a 0–10 scale. In addition, each child placed each picture in each series on a 100-cm long Visual Analogue Scale, with the anchors "not at all" and "a lot".

**Results:**

Children aged eight years or older rated the scales in the correct order 75% to 98% correctly, but children less than 8 years of age performed unreliably. The mean distance between each consecutive item in each pictorial scale was equal.

**Conclusion:**

Preliminary results revealed that children aged 8 to 18 years understood and used these three scales measuring throat closing, chest tightness, and effort appropriately. The scales appear to accurately measure the construct of breathlessness, at least at an interval level. Additional research applying these scales to clinical situations is warranted.

## Background

Dyspnea or breathlessness is a subjective phenomenon that can be perceived, regardless of the presence or absence of disease [1]. The measurement of the severity of dyspnea is challenging. The most commonly employed measure is the Borg scale and modifications thereof [2-4] even though it was initially designed to measure the effects of perceived exertion rather than dyspnea. The Borg scale has proven to be remarkably useful clinically as it correlates well with various physiologic parameters. The Borg scale uses simple, descriptive, adjectives such as slight, moderate, and severe in an open-ended scale that is, however, usually presented with numbers from 6–20 or 0–10. Studies in adults required a level of comprehension and considerable briefing of subjects, rendering these scales difficult to apply in children [5]. Throughout this paper, the word "children" is used to refer to both children and adolescents.

The other commonly used scale to rate degree of breathlessness is a Visual Analogue Scale (VAS) which usually consists of a 100-mm line with two anchors such as "not at all breathless" at one end and "maximally breathless" at the other [2,6]. Qualitative components of the sensory aspect, such as perceived "tightness" or "heaviness", which could be measured have not been seen as particularly useful for measuring severity of breathlessness, but may be related to the nature of the underlying disease [7,8]. Virtually no systematic use of the measurement of dyspnea severity has been made in children [9]. On the other hand, scales for measurement of magnitude of effort required to perform a task (such as exercise) have been described [10,11]. These measurements are particularly challenging in children because of the need for understanding and distinguishing the separate concepts of breathlessness and exertion, and differentiating the respective sensation from the affective response to it [12].

We sought to devise a pictorial scale that would encompass the full range of the perception of breathlessness by children. The aim was to develop a category scale or scales with at least ordinal properties. By intentionally avoiding an open magnitude scale like the Borg scale, it should be possible to use the scale to compare sensation within (e.g. before versus after intervention) and between individuals. Once the scales were devised, psychometric validation was undertaken to determine initial properties of the scales.

## Methods

The IWK Health Center Research Ethics Board approved this research, and all participants and/or their parents signed an informed consent or assent form.

Content validity of the pictorial scales was achieved by using three focus group sessions for children to tell us how they perceived dyspnea or breathlessness. One was attended by six children with asthma, aged 8–16 years; another by five children with cystic fibrosis, aged 12–19 years; and a third consisted of 14 healthy children with no history of chronic cardiopulmonary disease, aged 8–16 years. Altogether there were 13 boys and 12 girls. Children with asthma and cystic fibrosis were targeted as they represent the commonest chronic respiratory diseases in childhood. Each session was attended by a pediatric respirologist, an occupational therapist with experience in having children use drawings to express their feelings, and a graphic artist. The children and adolescents first were asked to recall instances when they experienced breathlessness, and what images were conjured up in their minds as a result. A roundtable discussion ensued, and the children and adolescents were then asked to draw one or more picture as best they could of what they thought breathlessness would look like, The children and adolescents also described different sensations of breathlessness or dyspnea.

In consultation with the investigators, the illustrator drew pictures based on the drawings and descriptions of the children and adolescents who had been in the focus groups. The final sets of pictures were determined by consensus among the investigators. Our selection of three scales was determined by the fact that some children drew and described throat constriction and some described and drew chest tightness. Many children also commented or drew pictures of effort, 'tired' or exhaustion. In each series, we coached the illustrator to draw pictures that we thought represented breathlessness of equal intervals from no breathlessness to maximal breathlessness.

**Figure 1:**
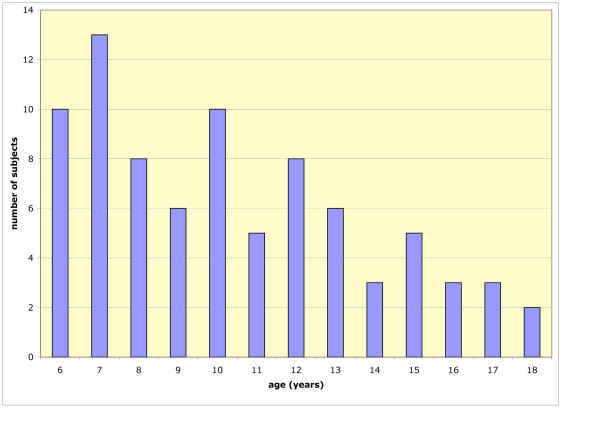
Age breakdown of the participants.

Initial psychometric validation and determination of scale properties was then carried out with 20 healthy controls (mean age 8.7 ± 2.4 years, 10 female), 29 children with asthma (mean age 10.4 ± 3.3 years, 18 female), and 30 children with cystic fibrosis (mean age 11.6 ± 3.6 years, 16 female). Figure 1 shows the age breakdown of the participants.

The back of each card was numbered such that the picture depicting no breathlessness was given a "1" and the picture depicting maximal breathlessness was given a "7".

Each child or adolescent completed three tasks on each set of pictures. The order of the sets was randomized. First of all, a set containing seven pictures was shuffled and the pictures randomly placed in a row in front of the child. Beginning with the left-most picture, the child was asked to rate each of the seven pictures one after another on a zero to ten scale, where zero meant not at all breathless and ten meant a whole lot of breathlessness. These ratings were done to determine whether the child was able to judge the magnitude of breathlessness represented by each picture independent of the pictures being in order. Secondly, the child was asked to lay the pictures out from left to right, in the order he or she felt represented the least to the most breathlessness. Finally, participants were asked to place each of the seven pictures on a one-metre long frame, and were asked to place the cards in relations to the severity of breathlessness using the verbal descriptors "not at all" and "a whole lot" as anchors at either end. A 100 cm ruler with 1 cm gradations was attached to the side of the frame facing the research assistant, hidden from the child. The placement recorded by the research assistant was the measurement on the ruler that lined up with a small mark on the centre of the back of the card. This allowed the research assistant to identify where the card had been placed on the VAS. In this manner, we were able to ascertain the ratings of each picture, ordinal properties of each scale (whether or not the cards were placed in rank order on the VAS) and interval properties (distance between cards).

Multivariate, between-subjects, analysis of variance tests were used to test for the main effects and interactions between age, gender, and diagnosis, on VAS placements for each pictorial set. Repeated measures analysis of variance was used to evaluate the distances along the 100 cm VAS line between each picture within a given set of seven.

## Results

### Development of the scales

Following the focus groups, all drawings were reviewed by the investigators and common themes were chosen for inclusion in the scale, based on the responses by children in each group. The common themes chosen were throat closing, chest tightness, and effort because these were the sub-constructs drawn and described in the focus groups.

Although negative affect was mentioned by the children, we decided not to include an affective scale as it was mentioned only in conjunction with physical sensations represented by the three constructs: throat constriction, chest tightness and exhaustion.

**Figure 2:**
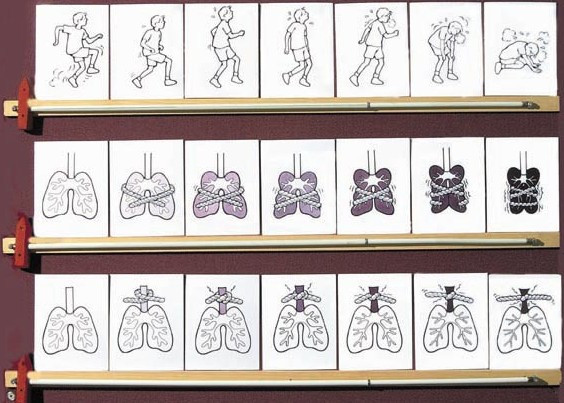
Dalhousie Dyspnea Scales.

The illustrator, with assistance from the investigators, integrated the drawings and verbal description of the children to develop illustrations that reflected what we had been told by the children and adolescents. For example, the ropes used in the figures were depicted as rough and heavy to convey the sensation which children described as sharp, burning, or needle-like and that several children and adolescents had portrayed using a rope or cord. We decided to use seven pictures of increasing severity in each series, as a compromise between providing sufficient resolution of degrees of breathlessness without overwhelming a viewer with too many choices. Once the drawings were completed, each scale consisted of seven individual pictures on laminated 12 × 15 cm cards. The final scales are shown in Figure 2.

### Evaluation of the scales

Multivariate, between-subjects, analysis of variance was used to test for the main effects and interactions between age, gender, and diagnosis, on VAS placements for each pictorial set. Repeated measures analysis of variance was used to test for significance the distances along the 100 cm VAS line between each picture within a given set of seven.

**Figure 3:**
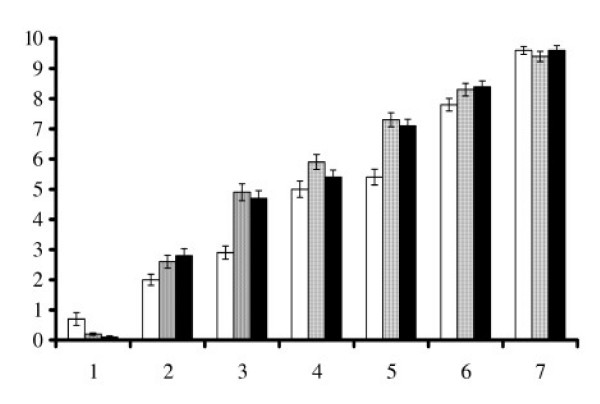
Mean Rating (SE) of breathlessness for each picture in each pictorial set.

Each of the three dyspnea scales (Throat closing, Chest tightness, and Effort) was initially rated on a 0 (none at all) to 10 (a whole lot) scale by each child. Figure 3 shows the mean ratings of breathlessness for each picture on the three scales, with the anchors 0 representing "no breathlessness at all", and 10 representing "the worst breathlessness imaginable". There was a consistent increase in ratings from the first to the seventh pictures.

Inspection of a scatter plot of the data revealed that there was a natural break, with 6 and 7 year old children rating in a less consistent manner than older children. We divided the sample into two age groups to better examine the performance of younger participants. Data from children aged six to seven years were grouped (n = 22, mean age 6.6 ± 5 years) and compared with the remaining participants aged 8–18 years (n = 56, mean age 12.0 ± 2.9 years). Table 1 shows the percentage of pictures correctly ranked by both age groups within each scale. The number of perfect rankings (i.e. all seven pictures within a set correctly ranked) was also investigated for each age group (Table 2). Since the ranking by 6–7 year old participants did not support the requirement that the scales have ordinal properties when employed in this age group, the data from 6 and 7 year olds were excluded from subsequent analysis.

**Table 1:** Percentage of children who correctly ranked individual pictures within each pictorial set. n = 22, 6–7 year olds, n = 56, 8–18 year olds.

Picture no.	Effort		Throat narrowing		Chest tightness	
	Age 6–7	Age 8–18	Age 6–7	Age 8–18	Age 6–7	Age 8–18

1	54.5	85.7	87.0	98.2	73.9	94.6
2	31.8	85.7	73.9	98.2	47.8	94.6
3	40.9	85.7	56.5	96.4	56.5	96.4
4	40.9	76.8	60.9	92.9	47.8	96.4
5	45.5	80.4	52.2	83.9	52.2	96.4
6	50.0	94.6	56.5	75.0	47.8	96.4
7	59.1	92.9	65.2	87.5	69.6	98.2

**Table 2:** Percentage of children who perfectly ranked individual pictures within each pictorial set. n = 22, 6–7 year olds, n = 56, 8–18 year olds.

Age group	Effort	Throat narrowing	Chest tightness
6–7	22	39	43
8–18	66	73	91

The remaining group of 56 participants comprised of 14 healthy controls (mean age 9.8 ± 2.1 years), 18 children with asthma (mean age 12.7 ± 2.4 years), and 24 children with cystic fibrosis (mean age 12.8 ± 2.9 years). Children with cystic fibrosis had mean (SD) FEV1 of 75 (22)% predicted. Our children with cystic fibrosis had the full spectrum of mild to severe of breathing difficulty. Children with asthma had mean (SD) FEV1 of 103% predicted, though we do not know how low their FEV1 dropped during exercise induced bronchoconstriction, or during flare-ups. Overall, they had mild to moderate disease.

**Figure 4:**
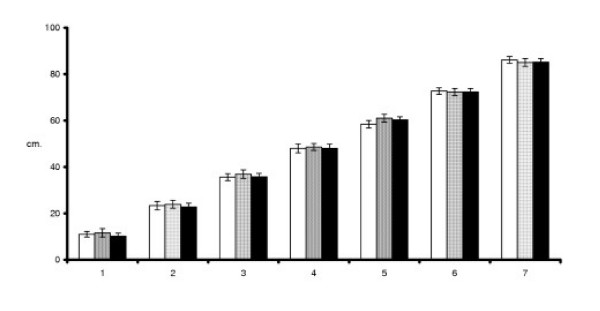
Mean (SE) VAS placements for each picture in each pictorial set

Figure 4 shows a gradual increase in the mean VAS placements when children were asked to place the pictures on a one-metre board. The mean distances between successively ranked pictures in each pictorial set were 12.5 ± 0.4 cm, 12.5 ± 1.2 cm, and 12.2 ± 0.5 cm respectively for the Chest tightness, Effort, and Throat closing pictorial scales. These distances between consecutive pictures were not significantly different.

Multivariate, between-subjects analysis of variance showed a main effect of underlying pulmonary disease on the sixth (*p *= .015) and seventh (*p *= .013) pictures in the Chest Tightness pictorial set. Children with asthma placed the sixth picture at 75.6 cm and the seventh picture at 89.7 cm along the VAS line. Children with cystic fibrosis and healthy children placed the sixth picture at 69.6 cm, 74.7 cm, and the seventh at 82.7 cm, 87.7 cm, respectively. No significant main effects or interactions were observed for the VAS placements of the Throat closing or Effort sets of pictures.

## Discussion

All three pictorial scales were understood by children over 8 years of age and were found to have at least interval properties and be valid psychophysical measures of self report of dyspnea in children with cystic fibrosis, asthma and in healthy children older than 8 years. Only some children less than 8 years of age used the pictures in a consistent way.

Our focus groups were conducted with children 8–19 years and the validation sample included children 6–18 years. We designed the scales for use of 8–19 year olds and then challenged the scales with younger children to see if the scales were robust in this age group. We believe that the constructs are difficult for children below 8 years to use. However, a useful scale might be developed for the younger age group.

The pictures in both the Chest tightness and Throat narrowing scales were rated and ranked more correctly than the items in the Effort pictorial scale. This may have been the result having more experience with, throat constriction or chest tightness when they experience dyspnea. In other words, the tightness images may have evoked better recall of the perceived intensity of sensation that the children experience during episodes of breathlessness. The sense of breathing effort, while related to dyspnea, can occur independently of difficulty breathing, and may require more cognitive complexity to understand. In this study, the Effort pictorial scale is not as robust as the other two scales and future research will determine if it needs to be revised.

The reasons why children with asthma placed the sixth and seventh pictures of the chest tightness scale differently than the normal children and the children with cystic fibrosis is not clear. We don't know if it is a reflection of true differences, or a quirk of the way this sample used the scale.

These scales were developed with Caucasian children in our centre in Canada. The results may not apply to other cultures that may conceptualize breathlessness in different ways. Moreover, children whose breathlessness is from causes other than cystic fibrosis and asthma such as children with vocal cord dysfunction or other upper airway problems may react differently. Similarly, we did not test children who suffer breathlessness because of lack of conditioning. Future studies should include these populations.

The Dalhousie Dyspnea Scales describe the sensation of breathing effort, as well as breathing difficulty. On the other hand, they do not measure the affective response [13]. This was purposefully avoided by showing only the lungs and throat, and not showing facial features on the Effort scale.

We did not control for multiple comparisons in our analyses. This was because, for the most part, the research questions were conceptually independent. Moreover our sample size was modest and a more conservative approach would reduce the power of the statistics used.

It will take further research to determine the best way to use the scales. For example, at this point, we do not know if combining the scales or if letting children use whichever scale is most appropriate to them yields the most meaningful data and the best scaling properties.

Our study did not correlate the self-report with physical measurements. These data speak only to the psychometrics of the self report of dyspnea. Future studies should examine how these scales relate to physical measures.

## Conclusion

The Dalhousie Dyspnea Scales were developed to ensure content validity and have been shown to have at least interval scale characteristics to measure breathlessness by children more than eight years old. Children readily use the scales with minimal instruction. Use of the scales in clinical studies and determination of the relationship between subjective breathlessness and objective physical stimuli is warranted.

## Abbreviations

Throughout this paper the term "children" is used to refer to children and adolescents up to 18 years of age.

## Competing interests

The author(s) declare that they have no competing interests.

## Authors' contributions

The study was conceived and designed by PJM & PTP with assistance from all remaining authors. The study was conducted under the supervision of PJM, PTP and conducted by CPB, PTP and AMU. Statistical analyses were conducted by PTP and CPB, with assistance from PJM. Interpretation of results were conducted by PJM, PTP, AMU. The manuscript was prepared by PJM and PTP and edited by AMU with review and assistance from all remaining authors. All authors read and approved the final manuscript.

## Pre-publication history

The pre-publication history for this paper can be accessed here:


